# *In vivo *efficacy of artemether-lumefantrine against uncomplicated *Plasmodium falciparum *malaria in Central Ethiopia

**DOI:** 10.1186/1475-2875-10-209

**Published:** 2011-07-28

**Authors:** Jimee Hwang, Bereket H Alemayehu, David Hoos, Zenebe Melaku, Samuel G Tekleyohannes, Takele Teshi, Sintayehu G Birhanu, Leykun Demeke, Kedir Gobena, Moges Kassa, Daddi Jima, Richard Reithinger, Henry Nettey, Michael Green, Joseph L Malone, S Patrick Kachur, Scott Filler

**Affiliations:** 1 U.S. Centers for Disease Control and Prevention, Atlanta, GA, USA; 2 Global Health Group, University of California San Francisco, CA, USA; 3 International Center for AIDS Care and Treatment Programs, Addis Ababa, Ethiopia; 4 International Center for AIDS Care and Treatment Programs, New York, USA; 5 Oromia Regional Health Bureau, Addis Ababa, Ethiopia; 6 Ethiopian Health and Nutrition Research Institute, Addis Ababa, Ethiopia; 7 Federal Ministry of Health, Addis Ababa, Ethiopia; 8 U.S. Agency for International Development, Addis Ababa, Ethiopia; 9 U.S. Centers for Disease Control and Prevention, Addis Ababa, Ethiopia; 10 The Global Fund to Fight AIDS, Tuberculosis and Malaria, Geneva, Switzerland

## Abstract

**Background:**

*In vivo *efficacy assessments of the first-line treatments for *Plasmodium falciparum *malaria are essential for ensuring effective case management. In Ethiopia, artemether-lumefantrine (AL) has been the first-line treatment for uncomplicated *P. falciparum *malaria since 2004.

**Methods:**

Between October and November 2009, we conducted a 42-day, single arm, open label study of AL for *P. falciparum *in individuals >6 months of age at two sites in Oromia State, Ethiopia. Eligible patients who had documented *P. falciparum *mono-infection were enrolled and followed according to the standard 2009 World Health Organization *in vivo *drug efficacy monitoring protocol. The primary and secondary endpoints were PCR uncorrected and corrected cure rates, as measured by adequate clinical and parasitological response on days 28 and 42, respectively.

**Results:**

Of 4426 patients tested, 120 with confirmed falciparum malaria were enrolled and treated with AL. Follow-up was completed for 112 patients at day 28 and 104 patients at day 42. There was one late parasitological failure, which was classified as undetermined after genotyping. Uncorrected cure rates at both day 28 and 42 for the per protocol analysis were 99.1% (95% CI 95.1-100.0); corrected cure rates at both day 28 and 42 were 100.0%. Uncorrected cure rates at day 28 and 42 for the intention to treat analysis were 93.3% (95% CI 87.2-97.1) and 86.6% (95% CI 79.1-92.1), respectively, while the corrected cure rates at day 28 and 42 were 94.1% (95% CI 88.2-97.6) and 87.3% (95% CI 79.9-92.7), respectively. Using survival analysis, the unadjusted cure rate was 99.1% and 100.0% adjusted by genotyping for day 28 and 42, respectively. Eight *P. falciparum *patients (6.7%) presented with *Plasmodium vivax *infection during follow-up and were excluded from the per protocol analysis. Only one patient had persistent parasitaemia at day 3. No serious adverse events were reported, with cough and nausea/vomiting being the most common adverse events.

**Conclusions:**

AL remains a highly effective and well-tolerated treatment for uncomplicated falciparum malaria in the study setting after several years of universal access to AL. A high rate of parasitaemia with *P. vivax *possibly from relapse or new infection was observed.

**Trial Registration:**

NCT01052584

## Background

Artemisinin-based combination therapy (ACT) has now been adopted by all countries in sub-Saharan Africa for the first-line treatment of uncomplicated *Plasmodium falciparum *malaria [1]. Routine monitoring of first-line therapies is crucial to ensure use of efficacious regimens and to maintain the progress made to date in decreasing malaria morbidity and mortality [2,3].

In Ethiopia, malaria is a leading cause of morbidity and mortality [1]. In 2007-2008, malaria was the most common cause of outpatient visits and admissions, accounting for 12% of all visits and 10% of admissions [4]. Unlike much of Africa, both *P. falciparum *and *Plasmodium vivax *contribute to malaria morbidity in Ethiopia, in relative proportions of 60% and 40%, respectively. However, this relative proportion varies both temporally and geographically, with published ranges of 22-89% for *P. falciparum *and 11-67% for *P. vivax *[5,6]. Furthermore, malaria affects all age groups.

Chloroquine (CQ)-resistant *P. falciparum *became a major public health threat in the early 1990s in Ethiopia [7]. By the late 1990s, 86-88% treatment failure rates with CQ were being reported, which prompted change of first-line treatment to sulphadoxine-pyrimethamine (SP) in 1998 [8,9]. In 2003, a nation-wide study evaluating SP efficacy showed 36% and 72% treatment failure rates with 14-day and 28-day follow-up, respectively [10]. Following a large-scale malaria epidemic that ravaged Ethiopia in 2003 [11] and the concomitant recognition of wide-spread resistance to SP [10,12], the Federal Ministry of Health (FMOH) adopted artemether-lumefantrine (AL) for first-line treatment of uncomplicated *P. falciparum *malaria in Ethiopia [1]. Prior to this change, baseline efficacy of AL was evaluated and a 1% treatment failure rate was noted [13]. More recent evaluations of AL efficacy in Ethiopia noted PCR uncorrected cure rates of 93% [14] and 96% [15]. AL is a blood schizonticide and has been shown to be effective, well-tolerated and fast-acting [16-18]. It was also the first co-formulated ACT meeting international good manufacturing practice standards and was pre-qualified by the World Health Organization (WHO).

In recent decades, spread of resistance to newly introduced anti-malarial therapies has been seen with dire consequences for malaria control. Reports of artemisinin resistance from the Thai-Cambodian border, the epicenter of drug resistance, raise global concern for the long-term efficacy of ACT [19,20]. Six years have passed since AL was adopted as first-line therapy in Ethiopia, which coincided with the FMOH's ambitious plan to provide universal access to prompt malaria diagnosis and treatment--as recently recommended by WHO [18] - through a network of 15,000 community-level health posts [21]. Continuously monitoring AL drug efficacy is critical to allow for sufficient time to explore alternatives and change national policy as soon as AL efficacy begins to decline.

The objectives of the study reported here were (i) to assess the *in vivo *efficacy of AL for *P. falciparum *at follow-up durations of 28 and 42 days, and (ii) to assess secondary outcomes of haematologic response, gametocytaemia, and rates of fever and parasite clearance.

## Methods

### Study site and enrollment

The study was conducted at two established FMOH health facilities, Bishoftu Malaria Clinic (elevation 1,850 metres) and Bulbula Health Center (elevation 2,150 metres), in Oromia Regional State, Ethiopia, from October to November, 2009. *Plasmodium falciparum *and *P. vivax *are endemic at both sites, and the study was conducted during the peak malaria transmission season. Malaria in Ethiopia is highly unstable, with potential for focal or large-scale epidemics. In Ethiopia, malaria transmission is largely determined by climate and altitude, with the peak transmission season occurring between September and December, after the main rainy season from June to August. Recent household surveys of all ages have noted parasite prevalence rates of 0.3 and 0.9% [21,22] while a school-based survey of children 5-18 years noted a prevalence of 0.6% [23] in Oromia, all consistent with hypoendemic transmission.

### Patients

Patients with *P. falciparum *mono-infection, with parasitaemia level of 1,000-100,000 asexual forms per μl were enrolled. Additional inclusion criteria included age >6 months, weight >5 kg, and axillary temperature ≥37.5°C or history of fever in the past 24 hours. Only patients living within 20 km of the enrolling study site were included to facilitate follow-up. Exclusion criteria included detection of any *Plasmodium *infection besides falciparum mono-infection, any signs or symptoms of severe illness, severe malnutrition, severe anaemia (haemoglobin (Hg) < 5 g/dL), known hypersensitivity to AL, presence of another infection or major co-morbid conditions, pregnancy, or breastfeeding.

The study received ethical clearance from the U.S. Centers for Disease Control and Prevention, Columbia University, and the Ethiopian Public Health Association. Written informed consent was obtained from adult patients and guardians of enrolled children. Written assent was also obtained from children 7-17 years of age.

### Clinical and laboratory procedures

To assess AL therapeutic efficacy for uncomplicated *P. falciparum *mono-infection based on clinical and parasitological parameters, a prospective, 42-day, open-label *in vivo *efficacy assessment according to the 2003 and 2009 WHO *in vivo *protocol for measuring anti-malarial drug efficacy for areas of low to moderate malaria transmission was conducted [3,24]. Blood films were taken at initial presentation for all patients at the Bishoftu Malaria Clinic (as routinely done). Once a positive *P. falciparum *infection was identified by microscopy, the patient was screened for study eligibility criteria. At Bulbula Health Center, all patients attending the outpatient ward were initially evaluated by the clinical staff and those with fever or a history of fever were referred for malaria testing (as routinely done). Once the patient was noted to have microscopy-confirmed *P. falciparum *infection, the study staff screened the patient for inclusion in the study.

After meeting the inclusion criteria and consenting to enrollment into the study, all patients underwent haemoglobin testing (Hemocue Hb 201+, Angelholm, Sweden) and filter paper blood spot collection. All women age 13-49 years underwent a urine pregnancy test and those testing positive were excluded from the study. Thick and thin blood films were prepared according to standard procedures, i.e. by staining for ten minutes with 10% Giemsa [24] and read by two independent, blinded microscopists. Parasite density was estimated based on the number of asexual parasites observed against 200 leukocytes assuming 8,000 leukocytes per μl. Patients with discrepant species results were excluded from the study. Slides with parasite densities differing by more than 20% between microscopists were reassessed by a third microscopist, with the third reading considered final. Smears were determined to be negative only after examining 100 high power fields.

Patients were interviewed and examined by the study clinician. The patients were given oral AL (Coartem^®^, Novartis Pharma, Basel, Switzerland) based on weight according to national policy [25]. AL was packaged in fixed-dose combination tablets, each containing 20 mg of artemether and 120 mg of lumefantrine. AL was administered according to the package insert twice daily for three days; those patients weighing 5-14 kg were given one tablet, 15-24 kg two tablets, 25-34 kg three tablets, and ≥35 kg four tablets at each of six dosing intervals. The initial and each morning dose were directly observed by the study staff and given with milk biscuits to increase absorption [26,27]. Patients were monitored for 60 minutes; a full or half a dose was re-administered if the patient vomited the drug within 30 or 31-60 minutes, respectively. If the patient vomited again, he or she was referred to the hospital and withdrawn from the study. Patients were instructed to take the evening dose with food. On follow-up days 1-3, patients were asked if the drug was taken properly the previous night. In order to facilitate twice daily dosing of AL, all patients presenting after 3:00 PM were excluded from the study. Antipyretics were given for temperatures > 39°C as per national policy [25]. Ferrous sulfate, folate, and mebendazole (if aged >1 year) were given to all children with haemoglobin <10 mg/dl as per Integrated Management of Childhood Illness (IMCI) guidelines [28]. Quinine (10 mg/kg every 8 hours for 7 days), the second-line treatment as per national policy, was administered as rescue therapy [25].

### Follow-up

Patients were followed-up for 42 days and asked to return on days 1-3, 7, 14, 21, 28, 35, and 42 post-AL therapy initiation, as well as any other interim day, if ill. The study site facilities were open from 8:00 AM to 6:00 PM, and after hours care was also available. Standardized follow-up included documentation of history-taking to elicit symptoms, adverse events, and any concomitant therapy, and physical examination including axillary temperature measurement. Finger pricks for follow-up blood films were taken on scheduled days 2, 3, 7, 14, 21, 28, 35, 42, and at any unscheduled visit. Haemoglobin was measured weekly and filter papers were collected on day 7 for drug level testing and on any day of failure for molecular testing. All filter papers were dried and stored in plastic storage bags with desiccant and humidity indicators. At the conclusion of the study, all day 0 slides were submitted to the national laboratory for quality control purposes. The filter papers for drug level testing were sent to the U.S. Centers for Disease Control and Prevention in Atlanta, USA, while the filter papers collected for molecular testing were sent to the Ethiopian Health and Nutrition Research Institute (EHNRI) in Addis Ababa, Ethiopia.

### Outcomes

Efficacy was assessed by clinical and parasitological outcomes using WHO definitions, with a 42-day follow-up period [3]. As per WHO definitions, early treatment failure (ETF) was defined as development of severe signs or symptoms, or insufficient parasitological response by day 3. Patients with *P. falciparum *parasitaemia occurring between 4 and 42 days without fever were classified as late parasitological failure (LPF) and those with fever as late clinical failure (LCF). If no failure was recorded, it was classified as adequate clinical and parasitological response (ACPR).

### Laboratory analysis

To distinguish between recrudescence and reinfection, three to four drops of blood were collected on filter paper at day 0 prior to treatment and on any day of recurrent parasitaemia. DNA was extracted from bloodspots dried on filter papers by soaking overnight in 1 ml of 0.5% saponin-1x phosphate buffered saline. The segment was then washed twice in 1 ml of PBS and boiled for 8 min in 100 μl PCR-grade water with 50 μl 20% chelex suspension (pH 9.5). Recrudescent and new infections were differentiated by typing the highly polymorphic repeat region of MSP2 as previously described [29]. Pre- and post-treatment samples for each patient were compared by running the amplified MSP2 products in adjacent lanes of 2% agarose gels. Recrudescent infections were characterized as having at least one identical allele (within 15 bp) present in both pre- and post-treatment samples. Samples where no alleles matched pre- and post-treatment were classified as new infections. If either pre- or post-samples failed to amplify, they were classified as undetermined. All molecular testing was conducted at EHNRI in Addis Ababa, Ethiopia.

Lumefantrine levels from day 7 dried blood spots were assessed in case of treatment failure. Lumefantrine levels in capillary whole blood were determined by high-performance liquid chromatography using a modified method described by Blessborn *et al *[30]. Briefly, lumefantrine was extracted from 8-mm diameter sections of dried blood-saturated filter paper using acetic acid and acetonitrile containing internal standard followed by solid-phase extraction. The filter papers were not treated with tartaric acid prior to blood application as suggested by Blessborn *et al *[30], resulting in lower recovery efficiency and a higher limit of quantification of 212 ng/ml. Chromatographic separation was accomplished using a Zorbax SB-CN, 3.0 × 250 mm, 5 micron column (Agilent Technologies, Santa Clara, CA, USA) with UV detection at 335 nm. Assay accuracy was determined to be 3.8% at 212 ng/ml and 11.4% at 5291 ng/ml.

### Statistical analysis

Primary efficacy outcomes included day 28 ACPR, both PCR corrected and uncorrected. Secondary outcomes included day 42 ACPR (PCR corrected and uncorrected), haematologic outcomes, and rates of fever and parasite clearance. With treatment failure of AL for *P. falciparum *being reported at ≤5%, 10% was chosen as the estimated therapeutic failure rate. Assuming an estimated ACPR of 90% and a 20% loss to follow-up at 42 days, a sample size of 120 subjects was determined to result in a 95% exact binomial confidence interval from 82.4%-95.1%.

All case record forms were pre-printed and organized into individual patient record folders. At the end of each clinic visit, the forms were checked by the nurse for completeness prior to discharging the patient. All patient records were reviewed for accuracy by the site supervisor. All data were entered into both Microsoft ACCESS (Microsoft, Redmond, WA, USA) database and the Microsoft Excel (Microsoft, Redmond, WA, USA) standard database developed by WHO [3], and analyzed using SAS 9.2 (SAS Institute, Cary, NC, USA). Data were double-entered and any discrepancies resolved by referring to the original paper form. Patients enrolled in the study erroneously and later found not to meet the inclusion criteria were excluded from all analyses. The intention-to-treat (ITT) analysis included all enrolled patients who met the inclusion criteria and took at least one full dose of AL. Patients lost to follow-up or withdrawn from the study were considered as failures. Per protocol (PP) analysis of outcomes included data for patients who had completed follow-up. Those patients lost to follow-up or withdrawn due to protocol violations were excluded from the PP analysis. In addition, the cumulative risk of failure using the Kaplan-Meier method was computed for 28- and 42-days of follow-up. For the Kaplan-Meier analysis, lost to follow-up, withdrawals, and parasitaemia with a different species were censored on the last day of follow-up. Comparisons between groups were made using χ^2 ^test or Fisher's exact test for categorical variables and the Student's t-test or Wilcoxon rank sum test (i.e. for non-parametric outcomes) for continuous variables. A two-sided p-value < 0.05 was considered statistically significant.

## Results

### Patients

**Figure 1:**
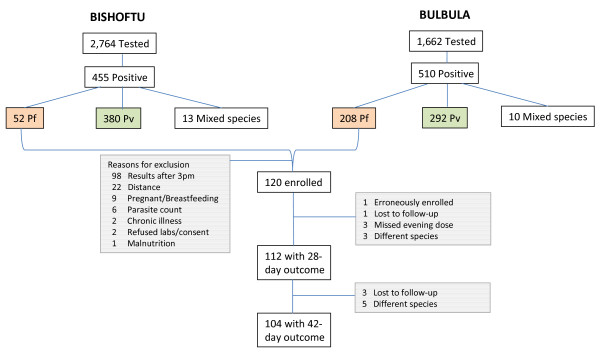
**Trial Profile**. Screening, Enrollment, and Follow-up of Patients

Of the 4,426 patients tested from the two study sites, 961 were positive for malaria parasites, and 260 tested positive for *P. falciparum *mono-infection. At the Bishoftu Malaria Clinic, the slide positivity rate was 16.5% (455/2,764) of which *P. falciparum *mono-infection accounted for only 11.4% (52/455); whereas, at Bulbula Health Center, the slide positivity rate was 30.7% (510/1,662) of which *P. falciparum *mono-infection accounted for 40.8% (208/510). In summary, 120 patients were enrolled. Trial profile and reasons for exclusion are shown in Figure 1. The majority of the excluded patients presented late in the afternoon and were not enrolled to ensure appropriate timing of the second dose of AL. One patient was incorrectly enrolled, with gametocytes only and no asexual parasites on day 0. This patient was withdrawn from the study and excluded from all analyses.

Baseline characteristics of the patients (N = 119) are shown in Table 1 stratified by children ≤5 years of age (n = 23) and those >5 years of age (n = 96). A significantly greater proportion of female patients were enrolled as children ≤5 years of age (47.8%) than in the group >5 years of age (20.8%) (p-value < 0.01). The geometric mean parasite density (GMPD) was significantly higher for children ≤5 years of age (27,198 parasites/μl; 95% confidence interval (CI) 19,468-37,996) than for those >5 years of age (14,500 parasites/μl; 95% CI 11,500-18,282) (p-value = 0.01). Furthermore, the presence of *P. falciparum *gametocytes at enrollment was significantly higher for those aged ≤5 years (26.1%) than those aged >5 years (8.3%) (p-value = 0.03). Although only 23.5% of patients reported owning a bed net of any type, 70.6% reported living in a household that had been sprayed with insecticide in the past 12 months. Bed net ownership and indoor residual spraying of households with insecticide did not differ significantly between the two age groups.

**Table 1:** Baseline characteristics of enrolled patients, stratified by age group under 5 years and over 5 years

Characteristic	≤5 years of age enrolled		>5 years of age enrolled		Total enrolled	
	
	n = 23	95% CI	n = 96	95% CI	N = 119	95% CI
Female (%)	47.8	26.8-69.4	20.8	13.2-30.3	26.1	18.4-34.9
Mean age (years)	3.1	2.6-3.6	19.9	17.4-22.5	16.7	14.3-19.1
Mean height (cm)	92.7	88.2-97.1	154.2	149.8-158.7	142.4	136.6-148.1
Mean weight (kg)	13.3	12.5-14.2	43.4	40.3-46.5	37.6	34.3-40.9
Mean temperature (°C)	37.6	37.1-38.1	37.5	37.3-37.7	37.5	37.3-37.7
Mean haemoglobin (g/dL)	10.4	9.6-11.1	13.2	12.7-13.6	12.6	12.2-13.1
Geometric Mean Parasite Density (parasites/μl)	27,198	19,468-37,996	14,500	11,500-18,282	16,374	13,375-20,045
Gametocytes present (%)	26.1	10.2-48.4	8.3	3.7-15.8	11.8	6.6-19.0
Owns a bed net (%)	21.7	7.5-43.7	24.0	15.8-33.8	23.5	16.2-32.2
IRS in past 12 months (%)	60.9	38.5-80.3	72.9	62.9-81.5	70.6	61.5-78.6

### Treatment outcomes

Follow-up was completed for 112 patients to day 28 and 104 to day 42 (Figure 1). There were no early treatment failures and only one late parasitological failure, which occurred on day 28 (Table 2). Molecular genotyping to distinguish recrudescence from reinfection for the one late parasitological treatment failure sample was classified as undetermined due to no amplification of the sample in both the pre- and post-treatment samples. This one late parasitological failure occurred in a 24-year-old male on day 28 of follow-up with parasite density of 3,840 parasites/μL and no symptoms.

**Table 2:** Treatment outcomes after day 28 and 42

	Duration of follow-up	
	
Outcome	Day 28	Day 42
	N = 119	N = 119
No treatment outcome	4 (3.4%)	7 (5.9%)
Lost to follow-up	1	4
Missed evening dose	3	3
Infection with different species	3 (2.5%)	8 (6.7%)
Failure		
Early Treatment Failure	0	0
Late Clinical Failure	0	0
Late Parasitological Failure	1	1
Adequate Clinical and Parasitological Response -uncorrected	111 (93.3%)	103 (86.6%)
Cure rates-Per Protocol, PCR uncorrected (95% CI)	99.1% (95.1-100.0)	99.0% (94.8-100.0)
Cure rates-Per Protocol, PCR corrected (95% CI)	100% (96.7-100.0)	100% (96.5-100.0)
Cure rates-Intention-to-treat, PCR uncorrected (95% CI)	93.3% (87.2-97.1)	86.6% (79.1-92.1)
Cure rates- Intention-to-treat, PCR corrected (95% CI)	94.1% (88.2-97.6)	87.3% (79.9-92.7)

Abbreviations: CI- confidence interval

Four patients were lost to follow-up and three withdrew from the study (after missing an evening dose of AL). The uncorrected cure rates at day 28 and 42 for the PP analysis were 99.1% (95% CI 95.1-100.0) and 99.0% (95% CI 94.8-100.0), respectively (Table 2). The corrected cure rates at day 28 and 42 for the PP analysis were 100.0% (95% CI 96.7-100.0) and 100.0% (95% CI 96.5-100.0), respectively (Table 2). The uncorrected cure rates at day 28 for the ITT analysis were 93.3% (95% CI 87.2-97.1) and 86.6% (95% CI 79.1-92.1), respectively (Table 2). The corrected cure rates at day 28 for the ITT analysis were 94.1% (95% CI 88.2-97.6) and 87.3% (95% CI 79.9-92.7), respectively (Table 2). Using survival analysis, the day 42 cure rate uncorrected and corrected by genotyping was 99.1% and 100.0%, respectively.

Eight patients (6.7%) were excluded from the PP analysis, when they presented with parasitaemia with a different species, *P. vivax *(Table 2). 50% (4/8) of the patients with *P. vivax *parasitaemia during follow-up were symptomatic at the time. The median age was 5 years with a range of 3-18 years and did not differ significantly for those that were symptomatic and those that were not. The parasite density for those that were symptomatic ranged from 120 to 80,640 parasite/μL, whereas for those without symptoms the range was 360 to 31,440 parasite/μL. The median time to appearance of parasitaemia with *P. vivax *was 35 days (interquartile range 28-37.5 days). Gender, age group, and presence of gametocytes on day 0 were explored as potential predictors of recurrent parasitaemia with a different species, but none were found to be significant.

**Figure 2:**
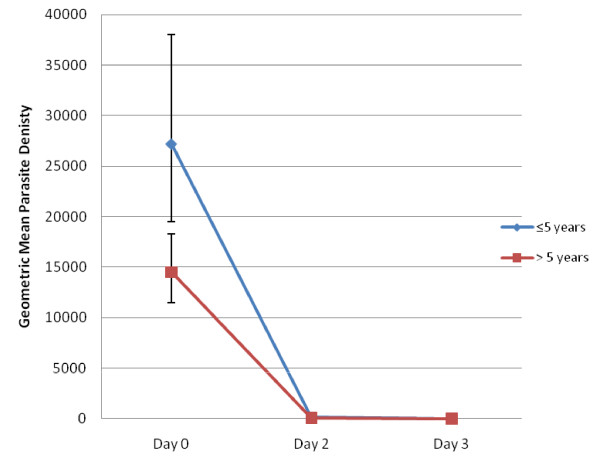
**Geometric Mean Parasite Density by day stratified by ≤5 years of age and >5 year of age with 95% confidence limits**.

Treatment with AL resulted in rapid clearance of parasites and resolution of fever. All but 8 (6.9%) patients had cleared their peripheral parasitaemia by day 2 and only one remained positive till day 3 (0.9%) (Table 3 and Figure 2). A review of parasite clearance data from several studies noted that positive blood smears on day 3 was found to be a good predictor of subsequent treatment failure and a simple screening measure for artemisinin resistance [31]. Children aged ≤ 5 years were more likely to have persistent parasitaemia on day 2 compared to those >5 years (p < 0.01). Of the 118 patients with fever (either by subjective report or measured axillary temperature ≥37.5°C) on day 0, 34.8% (n = 41) reported persistent fever on day 1 and only 7.0% (n = 8) by day 3. Restricting classification of fever to only those with recorded axillary temperature ≥37.5°C, 52.1% (62/119) were febrile on day 0 which decreased to only 8.5% (10/118) on day 1 and none by day 2. All patients with gametocytes on day 0 had persistent gametocytaemia on day 2. No gametocytes were noted after day 3 for those ≤5 years of age. After day 21, no gametocyte carriers were found in either age group. Excluding those with gametocytes on day 0, no new carriers were identified during the 42-day follow-up.

**Table 3:** Secondary outcomes stratified by under 5 years of age and over 5 years of age

Outcome	≤5 years of age	>5 years of age	Total
Fever*, n/N (%)			
Day 1	9/23 (39.1)	32/95 (33.7)	41/118 (34.8)
Day 2	1/22 (4.6)	10/94 (10.6)	11/116 (9.5)
Day 3	0/21 (0)	8/94 (8.5)	8/115 (7.0)
Parasitaemia, n/N (%)			
Day 2	5/22 (22.7)	3/94 (3.2)	8/116 (6.9)
Day 3	1/21 (4.8)	0/94 (0)	1/115 (0.9)
Gametocytaemia, n/N (%)			
Day 2	6/22 (27.3)	8/94 (8.5)	14/116 (12.1)
Day 3	1/21 (4.8)	5/94 (5.3)	6/115 (5.2)
Day 7	0	4/94 (4.3)	4/115 (3.5)
Day 14	0	1/94 (1.1)	1/115 (0.9)
Mean Haemoglobin g/dL, mean (SD)			
Day 28	12.0 (1.2)	13.7 (1.8)	13.4 (1.8)
Day 42	12.0 (1.1)	13.8 (1.8)	13.5 (1.8)
Mean Difference in Haemoglobin Day 0 to 42 g/dL, (SD)	1.9 (1.9)	0.7 (2.1)	0.9 (2.1)

*Subjective fever or axillary temperature ≥37.5°C

All day 0 slides submitted to the national laboratory at EHNRI for quality control showed the same species result except for two slides, which were diagnosed at the national laboratory as negative. Both blood smears were of very low level parasitaemia levels ranging from 1,240-1,640 parasites/μL and the possibility of an oversight at the national laboratory cannot be ruled out.

### Laboratory outcomes

Mean haemoglobin for all patients increased, from 12.6 g/dL (standard deviation (SD) 2.5) on day 0 to 13.5 g/dL (SD 1.8) on day 42. For children ≤5 years of age, it increased from 10.4 g/dL (SD 1.8) to 12.0 g/dL (SD 1.1) and for those >5 years of age from 13.2 g/dL (SD 2.3) to 13.8 g/dL (SD 1.8) (Table 2). The mean difference in haemoglobin levels between day 42 and 0 was 1.9 (SD 1.9) for those aged ≤5 years and 0.7 (SD 2.1) for those >5 years (p < 0.01 for both comparisons).

Whole blood capillary lumefantrine level testing from filter paper for the one treatment failure recovered 587.2 ng/mL of lumefantrine on day 7 with < 280 ng/ml previously identified to predict treatment failure.

### Adverse events

AL treatment was well tolerated. There were no serious adverse events. Four (3.5%) patients complained of cough, which was the most common complaint, followed by nausea and vomiting in three patients (2.6%).

## Discussion

This study showed that AL remains an efficacious and well-tolerated regimen in central Ethiopia. The high cure rates noted in this study are consistent with the baseline assessment conducted in 2003 prior to the scale-up of ACT [13], more recent studies in Ethiopia [14,15], and studies from elsewhere in Africa [17,32-36]. A recent meta-analysis of 32 published randomized studies of AL efficacy reported a 28-day PCR-corrected parasitological cure rate of 97% [17]. Depending on the methodology used, the cure rate for this study at day 28 ranged from 93-100% and at day 42 from 87-100%. Due to the very small number of failures, it was not possible to identify any predictors of treatment failures.

The rate of fever and parasite clearance was consistent with previous studies [37,38]. The study noted only one (0.9%) day 3 microscopically-positive *P. falciparum *case, after AL therapy who was ultimately classified as ACPR. The emerging resistance to artemisinins on the Thai-Cambodian border has been characterized by slow parasite clearance *in vivo *[19,20] and increased day 3 positive cases [19]. The review of parasite clearance times noted that artemisinin resistance was highly unlikely given the currently recommended 3-day ACT if the proportion of day 3 positive smears is <3% [31]. To date in Africa, no day 3 positives [37,38] to very few [39,40] [up to 1 percent] have been reported, and no resistance to AL has been reported [17]. However, with increasing parasite clearance times and proportion of cases positive on day 3 to artemisinin combination and monotherapies on the Thai-Cambodia border, efficacy studies of ACT should consider reporting parasite clearance times, or at least day 3 positivity rates, more consistently. Furthermore, the addition of day 1 blood films for all patients to our protocol, which was based on the standard WHO protocol, would have been easily accomplished and added another time point in defining the rate of parasite clearance.

The AL side-effects noted in this study are consistent with the ones previously reported [32,33,36,40,41]. A review of the safety profile of AL reported that most adverse events were mild or moderate in severity, common in both adults and children, and typical of symptoms of malaria [16]. Similar to this study, other studies from Africa have reported cough as the most common adverse event following AL therapy for *P. falciparum *infection [15,37]. However, the frequency was much lower in the current study, with only 3.5% patients experiencing cough compared to 29-46% in the other studies [15,37].

The study noted a significant improvement in haemoglobin from day 0 to 42. However, children with Hg < 10 g/dL received concomitant therapy with iron, folate, and mebendazole. A previous AL efficacy study from Ethiopia noted no significant Hg change after 28 days in the setting of no concomitant anaemia treatment and cited high rates of helminthic infections in the study area as a potential reason. Nonetheless, other efficacy studies conducted without concomitant anaemia treatment have noted significant improvement of haemoglobin levels [39,42,43].

Marked variability in drug absorption has been noted with lumefantrine administration [27] and the evaluation of lumefantrine levels have not been included in the standard WHO protocol although others have argued for measurement of day 7 drug level as a routine component of anti-malarial drug trials [44]. However, studies have reported plasma lumefantrine concentration of 280 ng/ml seven days from the start of treatment as a useful cut-off value for determining the risk of therapeutic failure [27,45]. Furthermore, capillary sampling was shown to be a suitable alternative to venous sampling [46]. Although, 587 ng/ml of lumefantrine was recovered from the one late parasitological failure on day 7 from filter paper, the methodology to consistently and accurately recover lumefantrine from filter paper samples is problematic. Although, the treatment of filter paper with tartaric acid prior to blood collection has been shown to improve recovery of lumefantrine [30], the utility of testing lumefantrine levels from filter paper to aid in accurately determining treatment failure remains challenging.

Recurrent parasitaemia with *P. vivax *during follow-up of *P. falciparum *efficacy studies have been reported from throughout Asia from Thailand [19,47,48], Cambodia [19], Papua [41], and Papua New Guinea [49], and more recently from Ethiopia [14,15]. The reason for the high rate of *P. vivax *parasitaemia found during follow-up, in general, is not clear, but the following hypotheses are possible: 1) initial co-infection where concurrent *P. falciparum *acutely suppresses *P. vivax *parasitaemia to levels below microscopic detection, 2) *P. falciparum *infection and its treatment might somehow activate dormant hypnozoites leading to *P. vivax *relapse, 3) new infection with *P. vivax *during the follow-up duration [50], or 4) activation of dormant hypnozoites is timed to seasonal peak transmission season coincident to a recently treated *P. falciparum *infection. Although the mechanism is unknown, the rate of subsequent *P. vivax *infection in the various studies noted lower rates when *P. falciparum *was treated with an effective drug with a longer half-life. For example, treatment of *P. falciparum *with dihydroartemisinin-piperaquine (piperaquine mean terminal elimination half-life is 28 days [51]) resulted in lower recurrent *P. vivax *compared to AL (lumefantrine mean terminal elimination half-life is 4.5 days [27]) [41,49]. The median time to recurrent parasitaemia with *P. vivax *in the study was 35 days after 42 days of follow-up, which presumably could have been longer had a longer acting, effective ACT been tested. The high rates of subsequent parasitaemia with *P. vivax *noted in this *P. falciparum in vivo *study, highlights the special challenges that *P. vivax *will pose for malaria control and elimination in Ethiopia and, indeed, the Horn of Africa.

Even with high efficacy of AL in Ethiopia, the effectiveness of this drug will not be optimally realized if patients are not accessing the drug in a timely manner or if patients do not adhere to the treatment regimen. The Malaria Indicator Survey from 2007 in Ethiopia noted that only 3.9% of children with fever were treated with an anti-malarial within 24 hours and of those treated, more received CQ than AL [52]. In an effort to improve supply of and access to free, prompt malaria diagnosis and treatment [21], Ethiopia has trained and deployed over 30,000 health extension workers to manage malaria at the community-level health posts. The impact of this large-scale deployment of AL was demonstrated in Tigray Regional State, where a two year study of community-based deployment of AL significantly lowered risk of malaria-specific mortality by 37% [2].

## Conclusions

Even after several years of free universal access to ACT, AL remains a highly efficacious treatment for uncomplicated *P. falciparum *infection in Ethiopia. The routine monitoring of first-line regimens is crucial for effective malaria control and national policy decision-making. In addition, some simple alterations of the current WHO protocol and reporting conventions could enhance a globally coordinated vigilance for early signals of declining efficacy of artemisinins.

## Competing interests

The authors declare that they have no competing interests.

## Authors' contributions

JH and BHA developed the protocol, supervised the study, conducted the analysis and drafted the manuscript. SPK and SF conceived of the study, assisted with the study design and protocol development, and helped draft the manuscript. ZM, SGT, DH, KG, MK, DJ, JLM, and RR participated in the study design, the study coordination and critically reviewed the draft manuscript. HN and MG carried out the drug level testing. SGB and LM supervised and conducted the laboratory analysis. TT supervised the study and assisted with data management. All authors read and approved the final manuscript.

## References

[B1] WHO World Malaria Report 2009 2009 Geneva: World Health Organization

[B2] Barnes KI Chanda P Ab Barnabas G Impact of the large-scale deployment of artemether/lumefantrine on the malaria disease burden in Africa: case studies of South Africa, Zambia and Ethiopia Malar J 2009 8 Suppl 1 S8 10.1186/1475-2875-8-S1-S8 19818175 PMC2760243

[B3] WHO Methods for surveillance of antimalarial drug efficacy 2009 Geneva: World Health Organization

[B4] Ethiopia Federal Ministry of Health Health and Health Related Indicators report EC 2000 2008 Addis Ababa: Ethiopia Federal Ministry of Health

[B5] Nigatu W Abebe M Dejene A *Plasmodium vivax *and *P. falciparum *epidemiology in Gambella, south-west Ethiopia Trop Med Parasitol 1992 43 181 185 1470839

[B6] Ramos JM Reyes F Tesfamariam A Change in epidemiology of malaria infections in a rural area in Ethiopia J Travel Med 2005 12 155 156 15996444 10.2310/7060.2005.12304

[B7] Alene GD Bennett S Chloroquine resistance of *Plasmodium falciparum *malaria in Ethiopia and Eritrea Trop Med Int Health 1996 1 810 815 8980594 10.1111/j.1365-3156.1996.tb00115.x

[B8] Kebede F Taffa N Tedla T An in-vivo study of falciparum malaria sensitivity to chloroquine in unstable malaria endemic area of central Ethiopia Ethiopian Medical Journal 1999 37 97 109 11957310

[B9] Tulu AN Webber RH Schellenberg JA Bradley DJ Failure of chloroquine treatment for malaria in the highlands of Ethiopia Trans R Soc Trop Med Hyg 1996 90 556 557 10.1016/S0035-9203(96)90322-3 8944273

[B10] Jima D Tesfaye G Medhin A Kebede A Argaw D Babaniyi O Efficacy of sulfadoxine-pyrimethamine for the treatment of uncomplicated falciparum malaria in Ethiopia East African Med J 2005 82 391 395 10.4314/eamj.v82i8.9322 16261914

[B11] Guthmann JP Bonnet M Ahoua L Dantoine F Balkan S van Herp M Tamrat A Legros D Brown V Checchi F Death rates from malaria epidemics, Burundi and Ethiopia Emerg Infect Dis 2007 13 140 143 10.3201/eid1301.060546 17370530 PMC2725810

[B12] Kassa M Sileshi M Mohammed H Taye G Asfaw M Development of resistance by *Plasmodium falciparum *to sulfadoxine/pyrimethamine in Amhara Region, Northwestern Ethiopia Ethiopian Medical Journal 2005 43 181 187 16370550

[B13] Jima D Tesfaye G Medhin A Kebede A Argaw D Babaniyi O Safety and efficacy of artemether-lumefantrine in the treatment of uncomplicated falciparum malaria in Ethiopia East African Med J 2005 82 387 390 10.4314/eamj.v82i8.9321 16261913

[B14] Kassa M *A study on efficacy of Coartem in the treatment of uncomplicated *Plasmodium falciparum *malaria in Shele, Arbaminch Zuria Wereda, South West Ethiopia* 2010 Addis Ababa: Ethiopian Health and Nutrition Research Institute

[B15] Assefa A Kassa M Tadese G Mohamed H Animut A Mengesha T Therapeutic efficacy of artemether/lumefantrine (Coartem^®^) against *Plasmodium falciparum *in Kersa, South West Ethiopia Parasit Vectors 2010 3 1 10.1186/1756-3305-3-1 20051120 PMC2881066

[B16] Falade C Manyando C Safety profile of Coartem: the evidence base Malar J 2009 8 Suppl 1 S6 10.1186/1475-2875-8-S1-S6 19818173 PMC2760241

[B17] Makanga M Krudsood S The clinical efficacy of artemether/lumefantrine (Coartem) Malar J 2009 8 Suppl 1 S5 10.1186/1475-2875-8-S1-S5 19818172 PMC2760240

[B18] WHO Guidelines for the treatment of malaria. Second edition 2010 Geneva: World Health Organization

[B19] Dondorp AM Nosten F Yi P Das D Phyo AP Tarning J Lwin KM Ariey F Hanpithakpong W Lee SJ Ringwald P Silamut K Imwong M Chotivanich K Lim P Herdman T An SS Yeung S Singhasivanon P Day NP Lindegardh N Socheat D White NJ Artemisinin resistance in *Plasmodium falciparum *malaria NEJM 2009 361 455 467 10.1056/NEJMoa0808859 19641202 PMC3495232

[B20] Noedl H Se Y Schaecher K Smith BL Socheat D Fukuda MM Evidence of artemisinin-resistant malaria in western Cambodia NEJM 2008 359 2619 2620 10.1056/NEJMc0805011 19064625

[B21] Jima D Getachew A Bilak H Steketee RW Emerson PM Graves PM Gebre T Reithinger R Hwang J Malaria indicator survey 2007, Ethiopia: coverage and use of major malaria prevention and control interventions Malar J 2010 9 58 10.1186/1475-2875-9-S2-P58 20178654 PMC2841196

[B22] Shargie EB Gebre T Ngondi J Graves PM Mosher AW Emerson PM Ejigsemahu Y Endeshaw T Olana D WeldeMeskel A Teferra A Tadesse Z Tilahun A Yohannes G Richards FO Malaria prevalence and mosquito net coverage in Oromia and SNNPR regions of Ethiopia BMC Public Health 2008 8 321 10.1186/1471-2458-8-321 18803880 PMC2556337

[B23] Ashton RA Kefyalew T Tesfaye G Pullan RL Yadeta D Reithinger R Kolaczinski JH Brooker S School-based surveys of malaria in Oromia Regional State, Ethiopia: a rapid survey method for malaria in low transmission settings Malar J 2011 10 25 10.1186/1475-2875-10-25 21288368 PMC3039636

[B24] WHO Assessment and monitoring of antimalarial drug efficacy for the treatment of uncomplicated falciparum malaria 2003 Geneva: World Health Organization

[B25] Ethiopia Federal Ministry of Health Malaria: diagnosis and treatment guidelines for health workers in Ethiopia 2nd Edition 2004 Addis Ababa: Federal Democratic Republic of Ethiopia, Ministry of Health

[B26] Ashley EA Stepniewska K Lindegardh N McGready R Annerberg A Hutagalung R Singtoroj T Hla G Brockman A Proux S Wilahphaingern J Singhasivanon P White NJ Nosten F Pharmacokinetic study of artemether-lumefantrine given once daily for the treatment of uncomplicated multidrug-resistant falciparum malaria Trop Med Int Health 2007 12 201 208 10.1111/j.1365-3156.2006.01785.x 17300626

[B27] Ezzet F van Vugt M Nosten F Looareesuwan S White NJ Pharmacokinetics and pharmacodynamics of lumefantrine (benflumetol) in acute falciparum malaria Antimicrob Agents Chemother 2000 44 697 704 10.1128/AAC.44.3.697-704.2000 10681341 PMC89749

[B28] WHO Handbook IMCI Integrated management of childhood illness 2006 Geneva: World Health Organization

[B29] Snounou G Zhu X Siripoon N Jarra W Thaithong S Brown KN Viriyakosol S Biased distribution of msp1 and msp2 allelic variants in *Plasmodium falciparum *populations in Thailand Trans R Soc Trop Med Hyg 1999 93 369 374 10.1016/S0035-9203(99)90120-7 10674079

[B30] Blessborn D Romsing S Annerberg A Sundquist D Bjorkman A Lindegardh N Bergqvist Y Development and validation of an automated solid-phase extraction and liquid chromatographic method for determination of lumefantrine in capillary blood on sampling paper J Pharm Biomed Anal 2007 45 282 287 10.1016/j.jpba.2007.07.015 17719735

[B31] Stepniewska K Ashley E Lee SJ Anstey N Barnes KI Binh TQ D'Alessandro U Day NP de Vries PJ Dorsey G Guthmann JP Mayxay M Newton PN Olliaro P Osorio L Price RN Rowland M Smithuis F Taylor WR Nosten F White NJ In vivo parasitological measures of artemisinin susceptibility J Infect Dis 2010 201 570 579 10.1086/650301 20085495 PMC4291277

[B32] Bakshi R Hermeling-Fritz I Gathmann I Alteri E An integrated assessment of the clinical safety of artemether-lumefantrine: a new oral fixed-dose combination antimalarial drug Trans R Soc Trop Med Hyg 2000 94 419 424 10.1016/S0035-9203(00)90126-3 11127248

[B33] Falade C Makanga M Premji Z Ortmann CE Stockmeyer M de Palacios PI Efficacy and safety of artemether-lumefantrine (Coartem) tablets (six-dose regimen) in African infants and children with acute, uncomplicated falciparum malaria Trans R Soc Trop Med Hyg 2005 99 459 467 10.1016/j.trstmh.2004.09.013 15837358

[B34] Faye B Ndiaye JL Tine R Sylla K Gueye A Lo AC Gaye O A randomized trial of artesunate mefloquine versus artemether lumefantrine for the treatment of uncomplicated *Plasmodium falciparum *malaria in Senegalese children Am J Trop Med Hyg 2010 82 140 144 10.4269/ajtmh.2010.09-0265 20065010 PMC2803524

[B35] Faye B Offianan AT Ndiaye JL Tine RC Toure W Djoman K Sylla K Ndiaye PS Penali L Gaye O Efficacy and tolerability of artesunate-amodiaquine (Camoquin plus) versus artemether-lumefantrine (Coartem) against uncomplicated *Plasmodium falciparum *malaria: multisite trial in Senegal and Ivory Coast Trop Med Int Health 2010 15 608 613 20214761 10.1111/j.1365-3156.2010.02487.x

[B36] Makanga M Premji Z Falade C Karbwang J Mueller EA Andriano K Hunt P De Palacios PI Efficacy and safety of the six-dose regimen of artemether-lumefantrine in pediatrics with uncomplicated *Plasmodium falciparum *malaria: a pooled analysis of individual patient data Am J Trop Med Hyg 2006 74 991 998 16760509

[B37] Dorsey G Staedke S Clark TD Njama-Meya D Nzarubara B Maiteki-Sebuguzi C Dokomajilar C Kamya MR Rosenthal PJ Combination therapy for uncomplicated falciparum malaria in Ugandan children: a randomized trial JAMA 2007 297 2210 2219 10.1001/jama.297.20.2210 17519410

[B38] Arinaitwe E Sandison TG Wanzira H Kakuru A Homsy J Kalamya J Kamya MR Vora N Greenhouse B Rosenthal PJ Tappero J Dorsey G Artemether-lumefantrine versus dihydroartemisinin-piperaquine for falciparum malaria: a longitudinal, randomized trial in young Ugandan children Clin Infect Dis 2009 49 1629 1637 10.1086/647946 19877969

[B39] Mulenga M VangGeertruyden JP Mwananyanda L Chalwe V Moerman F Chilengi R Van Overmeir C Dujardin JC D'Alessandro U Safety and efficacy of lumefantrine-artemether (Coartem) for the treatment of uncomplicated *Plasmodium falciparum *malaria in Zambian adults Malar J 2006 5 73 10.1186/1475-2875-5-73 16923176 PMC1579224

[B40] Sagara I Rulisa S Mbacham W Adam I Sissoko K Maiga H Traore OB Dara N Dicko YT Dicko A Jansen FH Doumbo OK Efficacy and safety of a fixed dose artesunate-sulphamethoxypyrazine-pyrimethamine compared to artemether-lumefantrine for the treatment of uncomplicated falciparum malaria across Africa: a randomized multi-centre trial Malar J 2009 8 63 10.1186/1475-2875-8-63 19366448 PMC2678145

[B41] Ratcliff A Siswantoro H Kenangalem E Maristela R Wuwung RM Laihad F Ebsworth EP Anstey NM Tjitra E Price RN Two fixed-dose artemisinin combinations for drug-resistant falciparum and vivax malaria in Papua, Indonesia: an open-label randomised comparison Lancet 2007 369 757 765 10.1016/S0140-6736(07)60160-3 17336652 PMC2532500

[B42] Sowunmi A Gbotosho GO Happi CT Adedeji AA Fehintola FA Folarin OA Tambo E Fateye BA Therapeutic efficacy and effects of artemether-lumefantrine and amodiaquine-sulfalene-pyrimethamine on gametocyte carriage in children with uncomplicated *Plasmodium falciparum *malaria in southwestern Nigeria Am J Trop Med Hyg 2007 77 235 241 17690392

[B43] Tshefu AK Gaye O Kayentao K Thompson R Bhatt KM Sesay SS Bustos DG Tjitra E Bedu-Addo G Borghini-Fuhrer I Duparc S Shin CS Fleckenstein L Efficacy and safety of a fixed-dose oral combination of pyronaridine-artesunate compared with artemether-lumefantrine in children and adults with uncomplicated *Plasmodium falciparum *malaria: a randomised non-inferiority trial Lancet 375 1457 1467 10.1016/S0140-6736(10)60322-4 20417857

[B44] White NJ Stepniewska K Barnes K Price RN Simpson J Simplified antimalarial therapeutic monitoring: using the day-7 drug level? Trends Parasitol 2008 24 159 163 10.1016/j.pt.2008.01.006 18353727

[B45] Ezzet F Mull R Karbwang J Population pharmacokinetics and therapeutic response of CGP 56697 (artemether + benflumetol) in malaria patients Br J Clin Pharmacol 1998 46 553 561 9862244 10.1046/j.1365-2125.1998.00830.x PMC1873796

[B46] van Vugt M Ezzet F Phaipun L Nosten F White NJ The relationship between capillary and venous concentrations of the antimalarial drug lumefantrine (benflumetol) Trans R Soc Trop Med Hyg 1998 92 564 565 10.1016/S0035-9203(98)90917-8 9861382

[B47] Ashley EA Krudsood S Phaiphun L Srivilairit S McGready R Leowattana W Hutagalung R Wilairatana P Brockman A Looareesuwan S Nosten F White NJ Randomized, controlled dose-optimization studies of dihydroartemisinin-piperaquine for the treatment of uncomplicated multidrug-resistant falciparum malaria in Thailand J Infect Dis 2004 190 1773 1782 10.1086/425015 15499533

[B48] Looareesuwan S White NJ Chittamas S Bunnag D Harinasuta T High rate of *Plasmodium vivax r*elapse following treatment of falciparum malaria in Thailand Lancet 1987 2 1052 1055 2889965 10.1016/s0140-6736(87)91479-6

[B49] Karunajeewa HA Mueller I Senn M Lin E Law I Gomorrai PS Oa O Griffin S Kotab K Suano P Tarongka N Ura A Lautu D Page-Sharp M Wong R Salman S Siba P Ilett KF Davis TM A trial of combination antimalarial therapies in children from Papua New Guinea NEJM 2008 359 2545 2557 10.1056/NEJMoa0804915 19064624

[B50] Douglas NM Anstey NM Angus BJ Nosten F Price RN Artemisinin combination therapy for vivax malaria Lancet Infect Dis 2010 10 405 416 10.1016/S1473-3099(10)70079-7 20510281 PMC3350863

[B51] Tarning J Ashley EA Lindegardh N Stepniewska K Phaiphun L Day NP McGready R Ashton M Nosten F White NJ Population pharmacokinetics of piperaquine after two different treatment regimens with dihydroartemisinin-piperaquine in patients with *Plasmodium falciparum *malaria in Thailand Antimicrob Agents Chemother 2008 52 1052 1061 10.1128/AAC.00955-07 18180343 PMC2258541

[B52] Ethiopia Federal Ministry of Health Ethiopia National Malaria Indicator Survey 2007 2008 Addis Ababa: Federal Ministry of Health, Ethiopia

